# Associations of hyperuricemia and obesity with remission of nonalcoholic fatty liver disease among Chinese men: A retrospective cohort study

**DOI:** 10.1371/journal.pone.0192396

**Published:** 2018-02-07

**Authors:** Chao Yang, Shujuan Yang, Chunhong Feng, Chuan Zhang, Weiwei Xu, Liyun Zhang, Yixin Yan, Jiaqi Deng, Okugbe Ebiotubo Ohore, Jing Li

**Affiliations:** 1 Department of Epidemiology and Health Statistics, School of Public Health, Southwest Medical University, Luzhou, China; 2 Department of Health Related Social and Behavioral Science, West China School of Public Health, Sichuan University, Chengdu, China; 3 Health Management Department, the First Affiliated Hospital, College of Medicine, Southwest Medical University, Luzhou, China; 4 Department of Palliative Medicine, No. 4 West China Teaching Hospital, Sichuan University, Chengdu, China; 5 Department of Ultrasonography, the First Affiliated Hospital, College of Medicine, Southwest Medical University, Luzhou, China; 6 Department of Public Health, Southeast University, Nanjing, China; 7 Department of Educational affairs, Southwest Medical University, Luzhou, China; Kaohsiung Medical University, TAIWAN

## Abstract

Nonalcoholic fatty liver disease (NAFLD) is a common chronic disease that is associated with high serum uric acid (SUA) levels, although the effects of high SUA levels on NAFLD remission remain unclear. In addition, it is unclear whether obesity and high SUA levels have a combined effect on NAFLD remission. This retrospective cohort study evaluated male employees of seven Chinese companies and investigated the association between high SUA levels and NAFLD remission, as well as the potential combined effect of high SUA levels and obesity on NAFLD remission. The study followed 826 men with NAFLD for 4 years, and the NAFLD remission rate was 23.2% (192/826). Comparing to obese and non-obese individuals with normouricemia, individuals with hyperuricemia had significant higher values for total cholesterol, triglycerides, creatinine, and aspartate transaminase (all *P* < 0.05). Among non-obese individuals, hyperuricemia was associated with a lower NAFLD remission rate, compared to normouricemia (*P* < 0.001). However, no significant difference was observed between hyperuricemia and normouricemia among obese subjects (*P* > 0.05). Similar results were observed in the multivariate cox proportional hazard regression analyses. Compared to the normouricemia subjects, individuals with hyperuricemia had a significant lower likelihood of NAFLD remission (RR = 0.535, 95% CI: 0.312–0.916); and obese subjects had a significant lower likelihood of NAFLD remission than the non-obese individuals (RR = 0.635, 95% CI: 0.439–0.918). In addition, the interaction between hyperuricemia and obesity had a statistically significant effect on NAFLD remission (*P* = 0.048). In conclusion, hyperuricemia and obesity may be involved in NAFLD development and remission, with similar pathogenic mechanisms. Further studies are needed to confirm our findings and determine how to improve these individuals’ conditions.

## Introduction

Nonalcoholic fatty liver disease (NAFLD) is the most prevalent chronic liver disease in Western and Asian countries, and approximately 20 million Chinese individuals (15% of the population) have NAFLD [1]. Recent studies have demonstrated that NAFLD precedes and is a risk factor for the development of metabolic syndrome [2], and patients with NAFLD have markedly higher risks of death from diabetes [3], cardiovascular disease, and liver-related disease [4], compared to the general population. Serum uric acid (SUA) is the end product of purine metabolism in the human body [5], as the endogenous precursors (nucleoproteins originating from cellular metabolism) and exogenous dietary precursors are delivered to the liver and subsequently excreted by the kidneys and intestine. Any disruption in this process can lead to high SUA levels [6, 7].

In 2002, a small Italian study revealed that SUA was closely associated with NAFLD [8], and subsequent evidence from three cohort studies [9–11] and two meta-analyses [12, 13] have confirmed that high SUA levels are independently associated with the development of NAFLD. However, few studies had focused on the relationship between NAFLD remission and high SUA levels. To the best of our knowledge, only one study of Chinese men has revealed that high baseline SUA levels were significantly associated with a lower likelihood of NAFLD remission [14]. Thus, additional evidence is needed to confirm the relationship between high SUA levels and NAFLD remission.

A community-based retrospective longitudinal cohort study has revealed that body mass index (BMI) can predict NAFLD onset [15]. Furthermore, several studies have evaluated the association between high SUA levels and the risk of NAFLD development among non-obese [16–19] and obese individuals [20, 21]. The vast majority of those studies consistently found that high SUA levels were independently associated with NAFLD risk, although a Brazilian study revealed that high levels of uric acid were not associated with NAFLD among overweight or obese children and adolescents [22]. Moreover, Zhang et al. have suggested that obesity and uric acid have a combined effect on the risk of NAFLD [23]. Therefore, we hypothesized that there might be an interactive effect of high SUA levels and obesity on the likelihood of NAFLD remission. As approximately 80% of individuals with NAFLD are male [24], the present study evaluated Chinese men to confirm the association between high SUA levels and NAFLD remission, and to explore the potentially interactive effect of high SUA levels and obesity on NAFLD remission.

## Materials and methods

### Ethical considerations

This study’s retrospective protocol was approved by the Ethics Committee of the First Affiliated Hospital (Clinical Medicine College, Southwest Medical University). Verbal informed consent had been obtained from each individual before they completed the physical examinations.

### Study design and subjects

**Fig 1:**
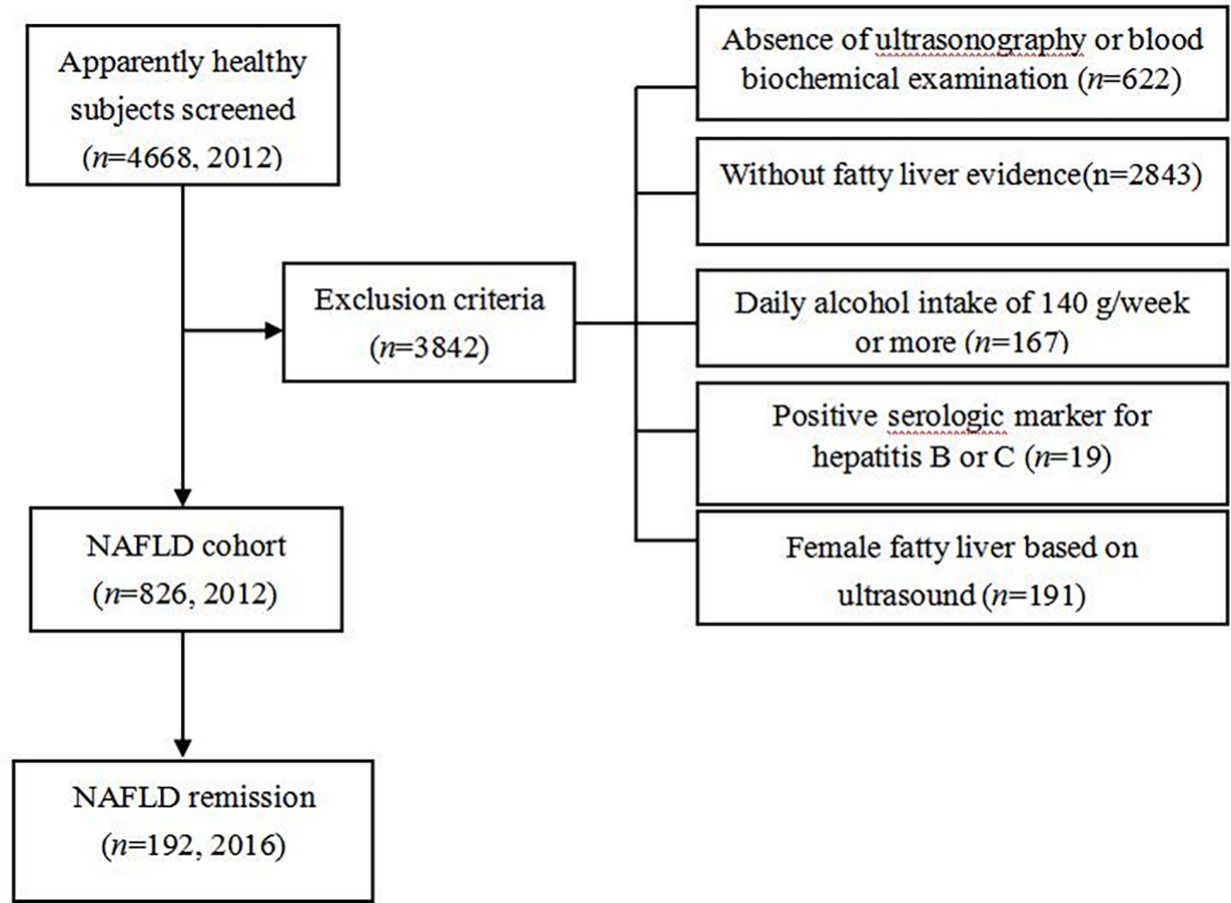
Flow diagram of subjects’s selection. A total of 4668 employees underwent health screening in 2012, of which 3842 subjects were excluded due to following reasons: absence of ultrasonography or blood biochemical examination; without fatty liver evidence; with heavy drinking(daily alcohol intake of 140g/week or more); with a positive serologic marker for hepatitis B or C; diagnosed with fatty liver based on ultrasonography in female; consequently, 826 subjects were observed for remission of NAFLD after 4 years.

This retrospective cohort study evaluated health examination data from 4,668 employees of seven Chinese companies. The examinations were performed in 2012 and 2016 at the first Affiliated Hospital of Southwest Medical University (Luzhou, China). A total of 3,842 subjects were excluded because of missing ultrasonography or blood biochemistry data, no evidence of fatty liver disease, heavy drinking (ethanol intake of ≥140 g/week), serological positivity for hepatitis B or C, or being women with fatty liver disease. Thus, the cohort included 826 men with NAFLD at the baseline examination, and these individuals completed the examination in 2016 to detect NAFLD remission (Fig 1).

### Measurements

The examinations included anthropometric measurements, hepatic ultrasonography, and biochemical testing. Systolic blood pressure (SBP) and diastolic blood pressure (DBP) were measured using an automated sphygmomanometer, with the subject in a seated position. Standing height and body weight were measured without shoes or heavy clothing, and BMI was reported as kg/m^2^. All examinations were performed between 8 AM and 11 AM after a 12-h overnight fast and before the subjects performed any exercise. The biochemical testing was performed using an automatic biochemical analyzer (SIEMENS ADVIA2400), which determined the levels of total cholesterol (TC), triglycerides (TG), high-density lipoprotein cholesterol (HDL-c), low-density lipoprotein cholesterol (LDL-c), fasting plasma glucose (FPG), creatinine (Cr), blood urea nitrogen (BUN), serum uric acid (SUA), aspartate aminotransferase (AST), and alanine aminotransferase (ALT).

### Outcomes and definitions

Based on the results of previous studies, we defined obesity as a BMI of ≥25 kg/m^2^ [16–19]. Hyperuricemia among men was defined as a SUA level of >420 μmol/L, while men with SUA levels at and below this cut-off were classified as having normouricemia [25]. We categorized the subjects into four groups based on their SUA levels and BMI: a group with normouricemia and normal weight (the **non-obese & normo** group), a group with hyperuricemia and normal weight (the **non-obese & hyper** group), a group with normouricemia and obesity (the **obese & normo** group), and a group with hyperuricemia and obesity (the **obese & hyper** group).

The diagnosis of fatty liver disease was based on results from abdominal ultrasonography (a GE Logig 9 sonography machine with a 10.0-MHz probe), which was performed by two senior imaging specialists. Hepatic steatosis was diagnosed based on the characteristic echo patterns from the conventional criteria, which include evidence of diffuse liver hyperechogenicity (vs. the kidneys), ultrasound beam attenuation, and poor visualization of the intrahepatic structures [26]. NAFLD was diagnosed based on with abdominal ultrasonography findings plus the exclusion of alcohol consumption, viral infection, or autoimmune liver disease [27].

### Statistical analyses

Continuous variables were expressed as mean ± standard deviation or median (interquartile range). Based on the data’s normality, variables were compared using Student’s t-test, one-way analysis of variance, or the Kruskal-Wallis H test, as appropriate. Categorical variables were compared using the chi-square test. Results from the multivariate Cox proportion regression analyses were expressed as relative risk (RR) and 95% confidence intervals (CIs). Model 1 analysed on hyperuricemia (hyper), obesity and the interaction of them (hyper*obese). Model 2 was adjusted for the variables in Model 1 plus age, SBP, DBP, ALT, AST, Cr, BUN, TC, TG, FPG, HDL-c, and LDL-c. Model 3 was adjusted for the variables in Model 2 plus the changes in SBP, DBP, Cr, BUN, SUA, TC, TG, FPG, HDL-c, LDL-c. All analyses were performed using SPSS software (version 17.0; SPSS Inc., Chicago, IL). All tests were two-tailed, and differences with a *P*-value of <0.05 were considered statistically significant.

## Results

### Baseline characteristics

At baseline, the 826 men had a mean age of 45.1 ± 10.8 years (range: 21–82 years). The baseline clinical characteristics of the four groups, according to their BMI and SAU levels, are presented in Table 1. Significant differences between the four groups were observed for BMI, SUA, TC, FPG, TG, HDL-c, BUN, Cr, ALT, AST, SBP, and DBP (all *P* < 0.05). However, no significant differences between the four groups were observed for age and LDL-c.

**Table 1:** Baseline characteristics of the non-obese & normo, non-obese & hyper, obese & normo, and obese & hyper groups.

	Non-obese individuals			Obese individuals			Overall *P*
	Normo (*n* = 242)	Hyper (*n* = 114)	*P*	Normo (*n* = 259)	Hyper (*n* = 211)	*P*	
Age (years)	46.5 ± 10.5	43.7 ± 11.2	0.021	44.8 ± 10.9	44.8 ± 10.8	0.985	0.088
BMI (kg/m^2^)	23.3 ± 1.4	23.3 ± 1.5	0.828	27.2 ± 1.8	27.7 ± 2.2	0.014	<0.001
SBP (mmHg)	129.1 ± 17.2	131.2 ± 16.8	0.279	131.9 ± 17.2	136.0 ± 20.7	0.020	0.001
DBP (mmHg)	85.8 ± 10.6	87.1 ± 10.9	0.288	88.3 ± 11.1	90.8 ± 13.2	0.031	<0.001
TC (mmol/L)	4.97 ± 1.00	5.28 ± 0.98	0.007	5.05 ± 0.92	5.32 ± 0.94	0.002	<0.001
TG (mmol/L)	1.95 (1.39–92.59)	2.43 (1.65–3.85)	<0.001	2.28 (1.47–3.04)	2.64 (1.88–3.78)	<0.001	<0.001
HDL-c (mmol/L)	1.27 ± 0.31	1.18 ± 0.34	0.018	1.16 ± 0.27	1.14 ± 0.25	0.317	<0.001
LDL-c (mmol/L)	2.62 ± 0.72	2.60 ± 0.76	0.837	2.69 ± 0.80	2.75 ± 0.80	0.444	0.205
FPG (mmol/L)	5.49 ± 1.72	5.37 ± 1.31	0.496	5.92 ± 2.18	5.59 ± 1.26	0.045	0.010
BUN (mmol/L)	5.33 ± 1.29	5.28 ± 1.37	0.724	5.56 ± 1.33	5.64 ± 1.50	0.570	0.027
Cr (μmol/L)	76.3 (69.1–83.7)	81.8 (70.5–89.0)	0.009	75.0 (69.3–84.4)	80.2 (74.0–91.7)	<0.001	<0.001
ALT (μmol/L)	28.3 (21.3–41.4)	35.9 (25.6–53.9)	<0.001	34.6 (25.9–50.2)	38.4 (25.7–54.9)	0.513	<0.001
AST (μmol/L)	26.4 (22.4–33.0)	30.5 (25.7–40.3)	<0.001	28.7 (24.1–36.2)	31.3 (25.3–38.1)	0.019	<0.001
SUA (mg/dL)	5.89 ± 0.78	8.06 ± 0.86	<0.001	6.07 ± 0.82	8.26 ± 0.92	<0.001	<0.001

Data are expressed as mean ± standard deviation or median (interquartile range). The subjects were grouped according to serum uric acid (SUA) levels and body mass index (BMI): non-obese & normo, non-obese & hyper, obese & normo, and obese & hyper. The four groups were compared using one-way analysis of variance if variables obey normal distribution or the Kruskal-Wallis H test if variable not obey normal distribution (TG, Cr, ALT, AST). Among non-obese or obese individuals, similarly, the hyper and normo groups were compared using Student’s t-test and the Mann-Whitney U test.

SBP, systolic blood pressure; DBP, diastolic blood pressure; TC, total cholesterol; TG, triglycerides; HDL-c, high-density lipoprotein cholesterol; LDL-c, low-density lipoprotein cholesterol; FPG, fasting plasma glucose; BUN, blood urea nitrogen; Cr, creatinine; AST, aspartate aminotransferase; ALT, alanine aminotransferase.

Among both obese and non-obese individuals, hyperuricemia was associated with significantly higher values for TC, TG, Cr, and AST, compared to normouricemia (all *P* < 0.05). No significant differences were observed in the LDL-c and BUN values according to SUA levels or BMI. Among non-obese individuals, hyperuricemia was associated with significantly lower values for age and HDL-c, and significantly higher ALT values, compared to normouricemia. These differences were not observed among obese individuals. Among obese individuals, hyperuricemia was associated with significantly higher values for BMI, SBP, and DBP, and significantly lower FPG values, compared to normouricemia (Table 1).

### Changes in the subjects’ characteristics

The changes in the subjects’ characteristics over the 4-year period were shown in Table 2 (2016 values minus 2012 values). Significant changes were only detected in the values for BMI, SUA, and Cr. In the normo and hyper groups, non-obese individuals exhibited a significant increase in BMI over the 4 years, although the BMI of obese individuals in these groups remained nearly unchanged. However, no significant differences in BMI were observed when we compared the hyper and normo groups, regardless of whether the individuals were obese or non-obese. Compared to the hyper group, the normo group exhibited a greater increase in SUA levels, and this difference was observed among both non-obese and obese individuals. Among obese individuals, the Cr decrease in the hyper group was significantly greater than that in the normo group, although no significant difference was observed among non-obese individuals.

**Table 2:** Changes in the characteristics of the non-obese & normo, non-obese & hyper, obese & normo, and obese & hyper groups.

	Non-obese individuals			Obese individuals			Overall *P*
	Normo (*n* = 242)	Hyper (*n* = 114)	*P*	Normo (*n* = 259)	Hyper (*n* = 211)	*P*	
BMI (kg/m^2^)	0.5 ± 1.6	0.8 ± 2.2	0.156	-0.1 ± 1.7	0.0 ± 1.8	0.859	<0.001
SBP (mmHg)	-0.7 ± 13.9	0.6 ± 18.3	0.495	1.1 ± 16.7	1.0 ± 17.8	0.957	0.631
DBP (mmHg)	0.7 ± 10.3	3.6 ± 12.1	0.022	1.2 ± 11.2	1.2 ± 12.7	0.993	0.163
TC (mmol/L)	-0.22 ± 0.78	-0.36 ± 0.80	0.115	-0.34 ± 0.74	-0.39 ± 1.12	0.572	0.177
TG (mmol/L)	-0.10 (-0.56, 0.38)	-0.10 (-0.87, 0.49)	0.732	-0.12 (-0.63, 0.42)	-0.18 (-0.96, 0.56)	0.462	0.714
HDL-c (mmol/L)	-0.10 ± 0.22	-0.14 ± 0.24	0.161	-0.11 ± 0.22	-0.08 ± 0.31	0.289	0.275
LDL-c (mmol/L)	0.32 ± 0.66	0.29 ± 0.71	0.688	0.23 ± 0.64	0.18 ± 0.70	0.444	0.149
FPG (mmol/L)	-0.30 ± 1.18	-0.21 ± 0.94	0.451	-0.29 ± 1.60	-0.09 ± 1.54	0.165	0.344
BUN (mmol/L)	-0.04 ± 1.38	-0.06 ± 1.32	0.921	-0.25 ± 1.20	-0.33 ± 1.60	0.545	0.084
Cr (μmol/L)	-1.1 (-6.1, 3.8)	-2.2 (-6.9, 2.5)	0.346	-0.9 (-7.2, 3.2)	-3.0 (-9.4, 2.3)	0.013	0.020
SUA (mg/dL)	0.007 ± 0.015	0.001 ± 0.019	0.001	0.003 ± 0.016	-0.005 ± 0.020	<0.001	<0.001

Data are expressed as mean ± standard deviation or median (interquartile range). The subjects were grouped according to serum uric acid (SUA) levels and body mass index (BMI): non-obese & normo, non-obese & hyper, obese & normo, and obese & hyper. The four groups were compared using one-way analysis of variance if variables obey normal distribution or the Kruskal-Wallis H test if variables not obey normal distribution (TG, Cr). Among non-obese or obese individuals, similarly, the hyper and normo groups were compared using Student’s t-test and the Mann-Whitney U test.

SBP, systolic blood pressure; DBP, diastolic blood pressure; TC, total cholesterol; FPG, fasting plasma glucose; TG, triglycerides; HDL-c, high-density lipoprotein cholesterol; LDL-c, low-density lipoprotein cholesterol; BUN, blood urea nitrogen; Cr, creatinine.

### The association of hyperuricemia and obesity with NAFLD remission

**Fig 2:**
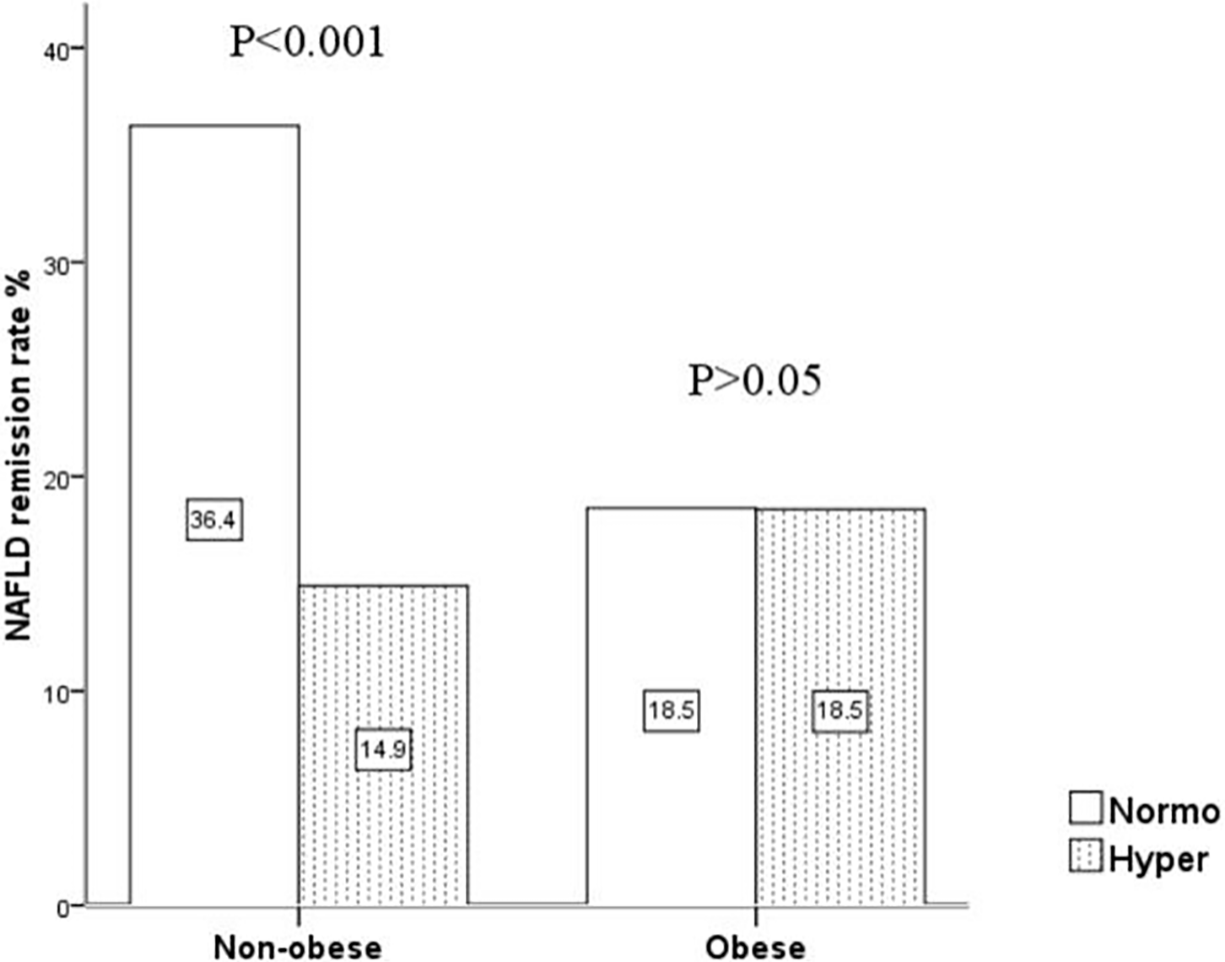
NAFLD remission rates of four groups (non-obese&normo, non-obese&hyper, obese&normo and obese&hyper). non-obese represents subjects with BMI<25; obese represents subjects with BMI≥25; hyper represents subjects with serum uric acid ≥420μmol/L; normo represents subjects with serum uric acid<420μmol/L. Hyper group had a lower NAFLD remission rate than that in normo group in non-obese subjects (P<0.001). However, there was no significant difference between hyper group and normo group in obese subjects (P>0.05).

The overall rate of NAFLD remission was 23.2% (192/826), with rates of 36.4% in the non-obese & normo group, 14.9% in the non-obese & hyper group, 18.5% in the obese & normo group, and 18.5% in the obese & hyper group (Fig 2). Individuals with hyperuricemia had a lower NAFLD remission rate, compared to individuals with normouricemia (*P* < 0.001), although no significant difference was observed between the hyper and normo groups among obese individuals (*P* > 0.05). The multivariate Cox proportional hazards regression analyses were used to estimate the effects of hyperuricemia, obesity, and the interaction of them on NAFLD remission. In model 1, hyperuricemia and obesity were both independent influencing factors for NAFLD remission. Moreover, the interaction of hyperuricemia and obesity had a single significant effect on NAFLD remission(*P* = 0.009). After adjusting for the baseline characteristics and their changes, the effects of the three factors on NAFLD remission were still statistically significant. Compared to the normouricemia subjects, individuals with hyperuricemia had a significant lower likelihood of NAFLD remission (RR = 0.535, 95% CI: 0.312–0.916); and obese subjects also had a significant lower likelihood of NAFLD remission than the non-obese individuals (RR = 0.635, 95% CI: 0.439–0.918). In addition, the interaction between hyperuricemia and obesity had a statistically significant effect on NAFLD remission (*P* = 0.048). (Table 3).

**Table 3:** The association of hyperuricemia and obesity with NAFLD remission.

variable	Model 1		Model 2		Model 3	
	*P*	*RR*(95%CI)	*P*	*RR*(95%CI)	*P*	*RR*(95%CI)
Hyper	0.001	0.410 (0.244–0.689)	0.006	0.479(0.283–0.811)	0.023	0.535(0.312–0.916)
Obese	<0.001	0.510(0.359–0.724)	0.006	0.601(0.418–0.865)	0.016	0.635(0.439–0.918)
Hyper*Obese	0.009	2.432(1.245–4.750)	0.028	2.130(1.084–4.184)	0.048	1.982(1.005–3.912)

Data are expressed as relative ratios and 95% confidence intervals for the multivariate Cox proportion regression analyses. Model 1 analysed on hyperuricemia (hyper), obese and the interaction of them (hyper*obese). Model 2 was adjusted for the variables in Model 1 plus age, systolic blood pressure, diastolic blood pressure, alanine transaminase, aspartate transaminase, serum creatinine, blood urea nitrogen, total cholesterol, triglycerides, fasting plasma glucose, high-density lipoprotein cholesterol, and low-density lipoprotein cholesterol. Model 3 was adjusted for the variables in Model 2 plus the changes in the study variables.

## Discussion

An increasing number of studies have confirmed that high SUA levels independently predict NAFLD, although the effects of high SUA levels on NAFLD remission remain unclear. The present study revealed that, among non-obese individuals, hyperuricemia was associated with a significantly lower NAFLD remission rate, compared to normouricemia. A previous study also reported similar findings, as elevated baseline SUA levels were significantly associated a lower rate of NAFLD remission at the end of the study [23]. Therefore, our study confirms the relationship between high SUA levels and NAFLD remission. Previous studies have also aimed to determine the mechanism through which high SUA levels are involved in NAFLD development. Lombardi et al. [28] have reviewed the accumulated evidence and suggested that SUA levels mainly contribute to NAFLD pathogenesis through their association with insulin resistance (IR) [29], radical oxygen species production [30], and activation of the Nod-like receptor pyrin domain-containing protein 3 (NLRP3) inflammasome [31]. In addition, Garcia-Ruiz et al. found that treating obese mice using uric acid led to a nearly complete resolution of fatty liver disease, which indicated that down-regulation of uric acid might play a protective role in NAFLD [32]. As suggested by Sun et al.’s meta-analysis [33], reduction of SUA levels may be a potential treatment for patients with NAFLD. However, those studies were unable to evaluate the biological mechanisms for the association of SUA levels with NAFLD in humans, as experimental animal models do not always mirror human biology. Thus, further clinical studies are needed to determine the therapeutic effect(s) of hyperuricemia attenuation in humans with NAFLD.

Obesity is closely associated with hyperuricemia, metabolic syndrome (MetS), and NAFLD, and some evidence indicates that obesity could modify the relationship between SUA and MetS. For example, a study of the general Korean population [34] revealed that SUA levels were associated with an increased risk of MetS in the subgroup with a BMI of <25 kg/m^2^, but not in the subgroup with a BMI of >25 kg/m^2^. However, the potential ability of obesity to modify the association of hyperuricemia with NAFLD remains unclear, as only one cross-sectional study of a large Chinese population has suggested that obesity and elevated uric acid levels have significant synergistic effects on the development of NAFLD [23]. Ours is the first study to evaluate whether hyperuricemia and obesity have a combined effect on NAFLD remission, and we found that there was no significant difference in the NAFLD remission rates of obese individuals with hyperuricemia or normouricemia. In addition, the interaction of hyperuricemia and obesity on NAFLD remission was observed in the multivariate cox proportional hazards regression models, after adjusted for baseline characteristics and their changes. Thus, rather than the expected synergistic effects (based on the previous study’s findings), we observed some form of an antagonistic effect.

This phenomenon has two possible explanations. First, obese subjects with hyperuricemia may be more sensitive to their own uric acid and body weight, which might have made them more likely to manage their uric acid levels and weight during the 4-year period, and the data in Table 2 support this explanation. For example, BMI increases were observed in the non-obese & normo and non-obese & hyper groups, while the BMI of obese individuals remained nearly unchanged. In addition, the SUA increase in the normo group was significant higher than that in the hyper group for non-obese and obese subjects. A small prospective cohort study has also revealed that weight gain and baseline IR predicted the incidence of NAFLD [35]. As the final multivariate model was adjusted for potential confounding, a second possible explanation is that hyperuricemia and obesity may be involved in NAFLD development and remission through similar pathogenic mechanisms. For example, both hyperuricemia and obesity are associated with IR, and Li et al. have suggested that elevated SUA levels may lead to IR development as a result of decreased endothelial nitric oxide bioavailability [29]. Obesity is also strongly linked to IR, which may be the result of hepatic IR caused by stimulation of the pro-inflammatory M1 macrophages in adipose tissues, which release interleukin-6, tumor necrosis factor-alpha [36], and free radicals that produce oxidative stress [8]. In addition, research in an African-American population [37] revealed that increases in abdominal subcutaneous and visceral adipose tissue were closely related to obesity and IR. Moreover, recent evidence indicates that the NLRP3 inflammasome can be directly activated by uric acid, and indirectly activated through radical oxygen species production [38]. Visceral adipose tissue can also induce an increased level of free fatty acids in the hepatic circulation, which leads to chronic low-grade inflammation in fatty liver disease [39]. Thus, subjects with hyperuricemia or obesity may be experiencing systemic inflammation.

Previous studies have also suggested that uric acid can cause mitochondrial oxidative stress [40], activation of sterol regulatory element-binding protein 1 after endoplasmic reticulum stress [41], and NLRP3 inflammasome activation [42], which can all negatively affect lipid metabolism. Interestingly, we observed that hyperuricemic subjects had significant higher TC and TG levels, compared to obese and non-obese normouricemic subjects (Table 2). However, obesity can also lead to various metabolic abnormalities, including abnormal lipid metabolism. The development of abdominal obesity is associated with an excess of cortisol [43], and the paracrine activity of cortisol in adipocytes may interfere with adipogenesis, adipocyte metabolism (*e*.*g*. increasing or decreasing lipolysis), and adipokine secretion [43]. Furthermore, these states do not exist alone and interact with other conditions. Thus, IR, systematic inflammation, and lipid metabolism disorders may represent an imbalanced environment that could be triggered by hyperuricemia and/or obesity. If this hypothesis is confirmed, it might be possible to treat NAFLD and induce remission by restoring balance to this internal environment. Further prospective studies are needed to determine whether lifestyle interventions (*e*.*g*. improving sleep, diet, exercise, and smoking habits) will be useful in this context.

The present study has several potential limitations. First, men and women have different physiological characteristics and behavior habits, Liu L et. Al[44] demostrated that lifestyles associated with male hyperuricemia was different with female. It was also been reported that gender has an impact on the association between hyperuricemia and MS risk[45]. Then, yang et.al [46] study on a cohort and found that the independent effect of hyperuricemia on NAFLD was stronger in females than in males. However, in the present study, we only evaluated Chinese men because of the small sample size of female NAFLD(191 subjects). Therefore, additional studies are needed to validate our findings among women and in broader populations. Second, the NAFLD diagnosis was based on ultrasonography findings, which may not reveal mild steatosis. Third, the retrospective design prevented us from collecting data regarding IR, waist circumstance, and dietary/lifestyle factors, which are associated with NAFLD. Thus, we could not assess their effect on NAFLD remission. Fourth, we considered individuals who were employed by specific companies, and it is possible that our findings may not be representative of the general Chinese population.

## Conclusions

This retrospective study confirmed that hyperuricemia is associated with NAFLD remission. Based on our findings, we hypothesize that hyperuricemia and obesity may be involved in NAFLD development and remission through similar pathogenic mechanisms. Further studies are needed to test our hypothesis and determine whether lifestyle interventions can address these mechanisms in individuals with these conditions.

## Supporting information

S1 Checklist STROBE_checklist_v4_combined_PlosMedicine. (DOCX)

S2 Checklist PLOSOne_Clinical_Studies_Checklist. (DOCX)

S1 Data Data. (RAR)

## References

[1] Wang FS, Fan JG, Zhang Z, Gao B, Wang HY. The global burden of liver disease: the major impact of China. Hepatology. 2014;60(6):2099–108. doi: 10.1002/hep.27406 ; PubMed Central PMCID: PMC4867229. 25164003 10.1002/hep.27406 PMC4867229

[2] Lonardo A, Ballestri S, Marchesini G, Angulo P, Loria P. Nonalcoholic fatty liver disease: a precursor of the metabolic syndrome. Digestive and liver disease: official journal of the Italian Society of Gastroenterology and the Italian Association for the Study of the Liver. 2015;47(3):181–90. doi: 10.1016/j.dld.2014.09.020 . 25739820 10.1016/j.dld.2014.09.020

[3] Zoppini G, Fedeli U, Gennaro N, Saugo M, Targher G, Bonora E. Mortality from chronic liver diseases in diabetes. The American journal of gastroenterology. 2014;109(7):1020–5. doi: 10.1038/ajg.2014.132 . 24890439 10.1038/ajg.2014.132

[4] Ekstedt M, Hagstrom H, Nasr P, Fredrikson M, Stal P, Kechagias S, et al Fibrosis stage is the strongest predictor for disease-specific mortality in NAFLD after up to 33 years of follow-up. Hepatology. 2015;61(5):1547–54. doi: 10.1002/hep.27368 . 25125077 10.1002/hep.27368

[5] Hediger MA, Johnson RJ, Miyazaki H, Endou H. Molecular physiology of urate transport. Physiology. 2005;20:125–33. doi: 10.1152/physiol.00039.2004 . 15772301 10.1152/physiol.00039.2004

[6] Mount DB, Kwon CY, Zandi-Nejad K. Renal urate transport. Rheumatic diseases clinics of North America. 2006;32(2):313–31, vi. doi: 10.1016/j.rdc.2006.02.006 . 16716882 10.1016/j.rdc.2006.02.006

[7] Richette P, Bardin T. Gout. Lancet. 2010;375(9711):318–28. doi: 10.1016/S0140-6736(09)60883-7 . 19692116 10.1016/S0140-6736(09)60883-7

[8] Angulo P. Nonalcoholic fatty liver disease. The New England journal of medicine. 2002;346(16):1221–31. doi: 10.1056/NEJMra011775 . 11961152 10.1056/NEJMra011775

[9] Xu C, Yu C, Xu L, Miao M, Li Y. High serum uric acid increases the risk for nonalcoholic Fatty liver disease: a prospective observational study. PLoS One. 2010;5(7):e11578 doi: 10.1371/journal.pone.0011578 ; PubMed Central PMCID: PMCPMC2904389. 20644649 10.1371/journal.pone.0011578 PMC2904389

[10] Lee JW, Cho YK, Ryan M, Kim H, Lee SW, Chang E, et al Serum uric Acid as a predictor for the development of nonalcoholic Fatty liver disease in apparently healthy subjects: a 5-year retrospective cohort study. Gut Liver. 2010;4(3):378–83. doi: 10.5009/gnl.2010.4.3.378 ; PubMed Central PMCID: PMCPMC2956352. 20981217 10.5009/gnl.2010.4.3.378 PMC2956352

[11] Xie Y, Wang M, Zhang Y, Zhang S, Tan A, Gao Y, et al Serum uric acid and non-alcoholic fatty liver disease in non-diabetic Chinese men. PLoS One. 2013;8(7):e67152 doi: 10.1371/journal.pone.0067152 ; PubMed Central PMCID: PMCPMC3720733. 23935829 10.1371/journal.pone.0067152 PMC3720733

[12] Zhou Y, Wei F, Fan Y. High serum uric acid and risk of nonalcoholic fatty liver disease: A systematic review and meta-analysis. Clinical biochemistry. 2016;49(7–8):636–42. doi: 10.1016/j.clinbiochem.2015.12.010 . 26738417 10.1016/j.clinbiochem.2015.12.010

[13] Wijarnpreecha K, Panjawatanan P, Lekuthai N, Thongprayoon C, Cheungpasitporn W, Ungprasert P. Hyperuricaemia and risk of nonalcoholic fatty liver disease: A meta-analysis. Liver international: official journal of the International Association for the Study of the Liver. 2016 doi: 10.1111/liv.13329 . 27891768 10.1111/liv.13329

[14] Zhou Z, Song K, Qiu J, Wang Y, Liu C, Zhou H, et al Associations between Serum Uric Acid and the Remission of Non-Alcoholic Fatty Liver Disease in Chinese Males. PLoS One. 2016;11(11):e0166072 doi: 10.1371/journal.pone.0166072 ; PubMed Central PMCID: PMC5106003. 27835657 10.1371/journal.pone.0166072 PMC5106003

[15] Miyake T, Kumagi T, Hirooka M, Furukawa S, Koizumi M, Tokumoto Y, et al Body mass index is the most useful predictive factor for the onset of nonalcoholic fatty liver disease: a community-based retrospective longitudinal cohort study. Journal of gastroenterology. 2013;48(3):413–22. doi: 10.1007/s00535-012-0650-8 . 22933183 10.1007/s00535-012-0650-8

[16] Chen CH, Huang MH, Yang JC, Nien CK, Yang CC, Yeh YH, et al Prevalence and risk factors of nonalcoholic fatty liver disease in an adult population of taiwan: metabolic significance of nonalcoholic fatty liver disease in nonobese adults. Journal of clinical gastroenterology. 2006;40(8):745–52. . 16940890 10.1097/00004836-200609000-00016

[17] Liu J, Xu C, Ying L, Zang S, Zhuang Z, Lv H, et al Relationship of serum uric acid level with non-alcoholic fatty liver disease and its inflammation progression in non-obese adults. Hepatology research: the official journal of the Japan Society of Hepatology. 2016 doi: 10.1111/hepr.12734 . 27172177 10.1111/hepr.12734

[18] Liu PJ, Ma F, Lou HP, Zhu YN, Chen Y. Relationship between serum uric acid levels and hepatic steatosis in non-obese postmenopausal women. Climacteric. 2014;17(6):692–9. doi: 10.3109/13697137.2014.926323 . 24884478 10.3109/13697137.2014.926323

[19] Cho HC. Prevalence and Factors Associated with Nonalcoholic Fatty Liver Disease in a Nonobese Korean Population. Gut Liver. 2016;10(1):117–25. doi: 10.5009/gnl14444 ; PubMed Central PMCID: PMCPMC4694743. 26260755 10.5009/gnl14444 PMC4694743

[20] Liu CQ, He CM, Chen N, Wang D, Shi X, Liu Y, et al Serum uric acid is independently and linearly associated with risk of nonalcoholic fatty liver disease in obese Chinese adults. Sci Rep. 2016;6:38605 doi: 10.1038/srep38605 ; PubMed Central PMCID: PMCPMC5141483. 27924915 10.1038/srep38605 PMC5141483

[21] Sartorio A, Del Col A, Agosti F, Mazzilli G, Bellentani S, Tiribelli C, et al Predictors of non-alcoholic fatty liver disease in obese children. European journal of clinical nutrition. 2007;61(7):877–83. doi: 10.1038/sj.ejcn.1602588 . 17151586 10.1038/sj.ejcn.1602588

[22] Cardoso AS, Gonzaga NC, Medeiros CC, Carvalho DF. Association of uric acid levels with components of metabolic syndrome and non-alcoholic fatty liver disease in overweight or obese children and adolescents. Jornal de pediatria. 2013;89(4):412–8. doi: 10.1016/j.jped.2012.12.008 . 23791233 10.1016/j.jped.2012.12.008

[23] Zhang S, Du T, Li M, Lu H, Lin X, Yu X. Combined effect of obesity and uric acid on nonalcoholic fatty liver disease and hypertriglyceridemia. Medicine (Baltimore). 2017;96(12):e6381 doi: 10.1097/MD.0000000000006381 ; PubMed Central PMCID: PMC5371466. 28328829 10.1097/MD.0000000000006381 PMC5371466

[24] Fan JG. Epidemiology of alcoholic and nonalcoholic fatty liver disease in China. Journal of gastroenterology and hepatology. 2013;28 Suppl 1:11–7. doi: 10.1111/jgh.12036 . 23855290 10.1111/jgh.12036

[25] Fang J, Alderman MH. Serum uric acid and cardiovascular mortality the NHANES I epidemiologic follow-up study, 1971–1992. National Health and Nutrition Examination Survey. Jama. 2000;283(18):2404–10. . 10815083 10.1001/jama.283.18.2404

[26] Targher G, Bertolini L, Poli F, Rodella S, Scala L, Tessari R, et al Nonalcoholic fatty liver disease and risk of future cardiovascular events among type 2 diabetic patients. Diabetes. 2005;54(12):3541–6. . 16306373 10.2337/diabetes.54.12.3541

[27] Jian-gao F, Chinese Liver Disease A. Guidelines for management of nonalcoholic fatty liver disease: an updated and revised edition. Zhonghua gan zang bing za zhi = Zhonghua ganzangbing zazhi = Chinese journal of hepatology. 2010;18(3):163–6. . 20698076

[28] Lombardi R, Pisano G, Fargion S. Role of Serum Uric Acid and Ferritin in the Development and Progression of NAFLD. Int J Mol Sci. 2016;17(4):548 doi: 10.3390/ijms17040548 ; PubMed Central PMCID: PMCPMC4849004. 27077854 10.3390/ijms17040548 PMC4849004

[29] Li C, Hsieh MC, Chang SJ. Metabolic syndrome, diabetes, and hyperuricemia. Current opinion in rheumatology. 2013;25(2):210–6. doi: 10.1097/BOR.0b013e32835d951e . 23370374 10.1097/BOR.0b013e32835d951e

[30] Zhu Y, Hu Y, Huang T, Zhang Y, Li Z, Luo C, et al High uric acid directly inhibits insulin signalling and induces insulin resistance. Biochemical and biophysical research communications. 2014;447(4):707–14. doi: 10.1016/j.bbrc.2014.04.080 . 24769205 10.1016/j.bbrc.2014.04.080

[31] Vandanmagsar B, Youm YH, Ravussin A, Galgani JE, Stadler K, Mynatt RL, et al The NLRP3 inflammasome instigates obesity-induced inflammation and insulin resistance. Nature medicine. 2011;17(2):179–88. doi: 10.1038/nm.2279 ; PubMed Central PMCID: PMC3076025. 21217695 10.1038/nm.2279 PMC3076025

[32] Garcia-Ruiz I, Rodriguez-Juan C, Diaz-Sanjuan T, del Hoyo P, Colina F, Munoz-Yague T, et al Uric acid and anti-TNF antibody improve mitochondrial dysfunction in ob/ob mice. Hepatology. 2006;44(3):581–91. doi: 10.1002/hep.21313 . 16941682 10.1002/hep.21313

[33] Sun DQ, Wu SJ, Liu WY, Lu QD, Zhu GQ, Shi KQ, et al Serum uric acid: a new therapeutic target for nonalcoholic fatty liver disease. Expert Opin Ther Targets. 2016;20(3):375–87. doi: 10.1517/14728222.2016.1096930 . 26419119 10.1517/14728222.2016.1096930

[34] Yu TY, Jee JH, Bae JC, Jin SM, Baek JH, Lee MK, et al Serum uric acid: A strong and independent predictor of metabolic syndrome after adjusting for body composition. Metabolism: clinical and experimental. 2016;65(4):432–40. doi: 10.1016/j.metabol.2015.11.003 . 26975535 10.1016/j.metabol.2015.11.003

[35] Zelber-Sagi S, Lotan R, Shlomai A, Webb M, Harrari G, Buch A, et al Predictors for incidence and remission of NAFLD in the general population during a seven-year prospective follow-up. Journal of hepatology. 2012;56(5):1145–51. doi: 10.1016/j.jhep.2011.12.011 . 22245895 10.1016/j.jhep.2011.12.011

[36] Schenk S, Saberi M, Olefsky JM. Insulin sensitivity: modulation by nutrients and inflammation. The Journal of clinical investigation. 2008;118(9):2992–3002. doi: 10.1172/JCI34260 ; PubMed Central PMCID: PMC2522344. 18769626 10.1172/JCI34260 PMC2522344

[37] Tulloch-Reid MK, Hanson RL, Sebring NG, Reynolds JC, Premkumar A, Genovese DJ, et al Both subcutaneous and visceral adipose tissue correlate highly with insulin resistance in african americans. Obesity research. 2004;12(8):1352–9. doi: 10.1038/oby.2004.170 . 15340119 10.1038/oby.2004.170

[38] Martinon F, Petrilli V, Mayor A, Tardivel A, Tschopp J. Gout-associated uric acid crystals activate the NALP3 inflammasome. Nature. 2006;440(7081):237–41. doi: 10.1038/nature04516 . 16407889 10.1038/nature04516

[39] Boden G, She P, Mozzoli M, Cheung P, Gumireddy K, Reddy P, et al Free fatty acids produce insulin resistance and activate the proinflammatory nuclear factor-kappaB pathway in rat liver. Diabetes. 2005;54(12):3458–65. . 16306362 10.2337/diabetes.54.12.3458

[40] Lanaspa MA, Sanchez-Lozada LG, Choi YJ, Cicerchi C, Kanbay M, Roncal-Jimenez CA, et al Uric acid induces hepatic steatosis by generation of mitochondrial oxidative stress: potential role in fructose-dependent and -independent fatty liver. The Journal of biological chemistry. 2012;287(48):40732–44. doi: 10.1074/jbc.M112.399899 ; PubMed Central PMCID: PMC3504786. 23035112 10.1074/jbc.M112.399899 PMC3504786

[41] Choi YJ, Shin HS, Choi HS, Park JW, Jo I, Oh ES, et al Uric acid induces fat accumulation via generation of endoplasmic reticulum stress and SREBP-1c activation in hepatocytes. Laboratory investigation; a journal of technical methods and pathology. 2014;94(10):1114–25. doi: 10.1038/labinvest.2014.98 . 25111690 10.1038/labinvest.2014.98

[42] Wan X, Xu C, Lin Y, Lu C, Li D, Sang J, et al Uric acid regulates hepatic steatosis and insulin resistance through the NLRP3 inflammasome-dependent mechanism. Journal of hepatology. 2016;64(4):925–32. doi: 10.1016/j.jhep.2015.11.022 . 26639394 10.1016/j.jhep.2015.11.022

[43] Ye J. Mechanisms of insulin resistance in obesity. Frontiers of medicine. 2013;7(1):14–24. doi: 10.1007/s11684-013-0262-6 ; PubMed Central PMCID: PMC3936017. 23471659 10.1007/s11684-013-0262-6 PMC3936017

[44] Liu L, Lou S, Xu K, Meng Z, Zhang Q, Song K. Relationship between lifestyle choices and hyperuricemia in Chinese men and women. Clinical rheumatology. 2013;32(2):233–9. doi: 10.1007/s10067-012-2108-z . 23132661 10.1007/s10067-012-2108-z

[45] Zhang J, Meng Z, Zhang Q, Liu L, Song K, Tan J, et al Gender impact on the correlations between subclinical thyroid dysfunction and hyperuricemia in Chinese. Clinical rheumatology. 2016;35(1):143–9. doi: 10.1007/s10067-015-2867-4 . 25875744 10.1007/s10067-015-2867-4

[46] Yang C, Yang S, Xu W, Zhang J, Fu W, Feng C. Association between the hyperuricemia and nonalcoholic fatty liver disease risk in a Chinese population: A retrospective cohort study. PLoS One. 2017;12(5):e0177249 doi: 10.1371/journal.pone.0177249 ; PubMed Central PMCID: PMC5433681. 28510581 10.1371/journal.pone.0177249 PMC5433681

