# Supplementary material for: Associations of hyperuricemia and obesity with remission of nonalcoholic fatty liver disease among Chinese men: A retrospective cohort study
Source: PLoS One. 2018 Feb 7;13(2):e0192396. doi: 10.1371/journal.pone.0192396 (PMC5802898; doi:10.1371/journal.pone.0192396)
Supplement: S1 Checklist — (DOCX) [file pone.0192396.s001.docx]

STROBE Statement—checklist of items that should be included in reports of observational studies

|  | Item No. | Recommendation | Page  No. | Relevant text from manuscript |
| --- | --- | --- | --- | --- |
| **Title and abstract** | 1 | (*a*) Indicate the study’s design with a commonly used term in the title or the abstract | 1 | A Retrospective Cohort Study |
|  |  | (*b*) Provide in the abstract an informative and balanced summary of what was done and what was found | 2 | **Abstract**  The study followed 826 men with NAFLD for 4 years, and the NAFLD remission rate was 23.2% (192/826).……Among non-obese individuals, hyperuricemia was associated with a lower NAFLD remission rate, compared to normouricemia (P < 0.001). However, no significant difference was observed between hyperuricemia and normouricemia among obese subjects (P > 0.05). |
| Introduction | | | |  |
| Background/rationale | 2 | Explain the scientific background and rationale for the investigation being reported | 4 | Nonalcoholic fatty liver disease (NAFLD) is the most prevalent chronic liver disease in Western and Asian countries, and approximately 20 million Chinese individuals (15% of the population) have NAFLD. Many studies have confirmed that high SUA levels are independently associated with the development of NAFLD. However, few studies had focused on the relationship between NAFLD remission and high SUA levels. |
| Objectives | 3 | State specific objectives, including any prespecified hypotheses | 5 | the present study evaluated Chinese men to confirm the association between high SUA levels and NAFLD remission, and to explore the potentially interactive effect of high SUA levels and obesity on NAFLD remission. |
| Methods | | | |  |
| Study design | 4 | Present key elements of study design early in the paper | 5 | **Study design and subjects** |
| Setting | 5 | Describe the setting, locations, and relevant dates, including periods of recruitment, exposure, follow-up, and data collection | 5,6 | This retrospective cohort study evaluated health examination data from 4,668 employees of seven Chinese companies. The examinations were performed in 2012 and 2016 at the first Affiliated Hospital of Southwest Medical University (Luzhou, China). A total of 3,842 subjects were excluded because of missing ultrasonography or blood biochemistry data, no evidence of fatty liver disease, heavy drinking (ethanol intake of ≥140 g/week), serological positivity for hepatitis B or C, or being women with fatty liver disease. Thus, the cohort included 826 men with NAFLD at the baseline examination, and these individuals completed the examination in 2016 to detect NAFLD remission (Fig 1). |
| Participants | 6 | (*a*) *Cohort study*—Give the eligibility criteria, and the sources and methods of selection of participants. Describe methods of follow-up  *Case-control study*—Give the eligibility criteria, and the sources and methods of case ascertainment and control selection. Give the rationale for the choice of cases and controls  *Cross-sectional study*—Give the eligibility criteria, and the sources and methods of selection of participants | 5,6 | This retrospective cohort study evaluated health examination data from 4,668 employees of seven Chinese companies. The examinations were performed in 2012 and 2016 at the first Affiliated Hospital of Southwest Medical University (Luzhou, China). A total of 3,842 subjects were excluded because of missing ultrasonography or blood biochemistry data, no evidence of fatty liver disease, heavy drinking (ethanol intake of ≥140 g/week), serological positivity for hepatitis B or C, or being women with fatty liver disease. Thus, the cohort included 826 men with NAFLD at the baseline examination, and these individuals completed the examination in 2016 to detect NAFLD remission (Fig 1). |
|  |  | (*b*)*Cohort study*—For matched studies, give matching criteria and number of exposed and unexposed  *Case-control study*—For matched studies, give matching criteria and the number of controls per case |  |  |
| Variables | 7 | Clearly define all outcomes, exposures, predictors, potential confounders, and effect modifiers. Give diagnostic criteria, if applicable | 6 | **Outcomes and definitions** |
| Data sources/measurement | 8* | For each variable of interest, give sources of data and details of methods of assessment (measurement). Describe comparability of assessment methods if there is more than one group | *6* | ***Baseline examinations*** |
| Bias | 9 | Describe any efforts to address potential sources of bias |  |  |
| Study size | 10 | Explain how the study size was arrived at |  |  |

| Quantitative variables | 11 | Explain how quantitative variables were handled in the analyses. If applicable, describe which groupings were chosen and why | 7 | Continuous variables were expressed as mean ± standard deviation or median (interquartile range). Based on the data’s normality, variables were compared using Student’s t-test, one-way analysis of variance, or the Kruskal-Wallis H test, as appropriate. |
| --- | --- | --- | --- | --- |
| Statistical methods | 12 | (*a*) Describe all statistical methods, including those used to control for confounding | 7 | **Statistical analyses** |
|  |  | (*b*) Describe any methods used to examine subgroups and interactions | 7 | **Statistical analyses** |
|  |  | (*c*) Explain how missing data were addressed |  |  |
|  |  | (*d*) *Cohort study*—If applicable, explain how loss to follow-up was addressed  *Case-control study*—If applicable, explain how matching of cases and controls was addressed  *Cross-sectional study*—If applicable, describe analytical methods taking account of sampling strategy |  |  |
|  |  | (*e*) Describe any sensitivity analyses |  |  |
| Results | | | | |
| Participants | 13* | (a) Report numbers of individuals at each stage of study—eg numbers potentially eligible, examined for eligibility, confirmed eligible, included in the study, completing follow-up, and analysed |  |  |
|  |  | (b) Give reasons for non-participation at each stage | 5 | **Study design and subjects** |
|  |  | (c) Consider use of a flow diagram | 5 | Fig 1 |
| Descriptive data | 14* | (a) Give characteristics of study participants (eg demographic, clinical, social) and information on exposures and potential confounders | 9 | **Baseline characteristics table 1** |
|  |  | (b) Indicate number of participants with missing data for each variable of interest | 10,11 | **Changes in the subjects’ characteristics table 2** |
|  |  | (c) *Cohort study*—Summarise follow-up time (eg, average and total amount) |  |  |
| Outcome data | 15* | *Cohort study*—Report numbers of outcome events or summary measures over time | 12 | The overall rate of NAFLD remission was 23.2% (192/826), with rates of 36.4% in the non-obese&normo group, 14.9% in the non-obese&hyper group, 18.5% in the obese&normo group, and 18.5% in the obese&hyper group (Fig 2). |
|  |  | *Case-control study—*Report numbers in each exposure category, or summary measures of exposure |  |  |
|  |  | *Cross-sectional study—*Report numbers of outcome events or summary measures |  |  |
| Main results | 16 | (*a*) Give unadjusted estimates and, if applicable, confounder-adjusted estimates and their precision (eg, 95% confidence interval). Make clear which confounders were adjusted for and why they were included | 12 | After adjusting for the baseline characteristics and their changes, and using the non-obese&normo group as the reference group, the RR values for NALFD remission were 0.553 (95% CI: 0.319–0.957) in the non-obese&hyper group, 0.580 (95% CI: 0.398–0.845) in the obese&normo group, and 0.593 (95% CI: 0.379–0.928) in the obese&hyper group (Table 3). |
|  |  | (*b*) Report category boundaries when continuous variables were categorized |  |  |
|  |  | (*c*) If relevant, consider translating estimates of relative risk into absolute risk for a meaningful time period |  |  |

Continued on next page

| Other analyses | 17 | Report other analyses done—eg analyses of subgroups and interactions, and sensitivity analyses | 13 | After adjusting for the baseline characteristics and their changes, and using the obese&hyper group as the reference group, the RR values for NAFLD remission were 1.752 (95% CI: 1.158–2.649) in the non-obese&normo group, 1.067 (95% CI: 0.645–1.764) in the non-obese&hyper group, and 1.032 (95% CI: 0.674–1.581) in the obese&normo group. |
| --- | --- | --- | --- | --- |
| Discussion | | | | |
| Key results | 18 | Summarise key results with reference to study objectives | 15 | Therefore, our study confirms the relationship between high SUA levels and NAFLD remission. |
| Limitations | 19 | Discuss limitations of the study, taking into account sources of potential bias or imprecision. Discuss both direction and magnitude of any potential bias | 18 | The present study has several potential limitations. First, the NAFLD diagnosis was based on ultrasonography findings, which may not reveal mild steatosis. Second, the retrospective design prevented us from collecting data regarding IR, waist circumstance, and dietary/lifestyle factors, which are associated with NAFLD. Thus, we could not assess their effect on NAFLD remission. Third, we only evaluated Chinese men, and additional studies are needed to validate our findings among women and in broader populations. Fourth, we considered individuals who were employed by specific companies, and it is possible that our findings may not be representative of the general Chinese population. |
| Interpretation | 20 | Give a cautious overall interpretation of results considering objectives, limitations, multiplicity of analyses, results from similar studies, and other relevant evidence | 18 | This retrospective study confirmed that hyperuricemia is associated with NAFLD remission. Based on our findings, we hypothesize that hyperuricemia and obesity may be involved in NAFLD development and remission through similar pathogenic mechanisms. Further studies are needed to test our hypothesis and determine whether lifestyle interventions can address these mechanisms in individuals with these conditions. |
| Generalisability | 21 | Discuss the generalisability (external validity) of the study results |  |  |
| Other information | |  | | |
| Funding | 22 | Give the source of funding and the role of the funders for the present study and, if applicable, for the original study on which the present article is based |  |  |

*Give information separately for cases and controls in case-control studies and, if applicable, for exposed and unexposed groups in cohort and cross-sectional studies.

**Note:** An Explanation and Elaboration article discusses each checklist item and gives methodological background and published examples of transparent reporting. The STROBE checklist is best used in conjunction with this article (freely available on the Web sites of PLoS Medicine at http://www.plosmedicine.org/, Annals of Internal Medicine at http://www.annals.org/, and Epidemiology at http://www.epidem.com/). Information on the STROBE Initiative is available at www.strobe-statement.org.
